# Over-dispersed *Trypanosoma cruzi* parasite load in sylvatic and domestic mammals and humans from northeastern Argentina

**DOI:** 10.1186/s13071-022-05152-7

**Published:** 2022-01-24

**Authors:** Gustavo Fabián Enriquez, Jacqueline Bua, María Marcela Orozco, Natalia Paula Macchiaverna, Julián Antonio Alvarado Otegui, Hernán Darío Argibay, María del Pilar Fernández, Ricardo Esteban Gürtler, Marta Victoria Cardinal

**Affiliations:** 1 Laboratorio de Eco-Epidemiología, Departamento de Ecología, Genética y Evolución, Facultad de Ciencias Exactas y Naturales, Universidad de Buenos Aires, Intendente Güiraldes 2160, Piso 2, Ciudad Universitaria, Buenos Aires, Argentina; 2 Instituto de Ecología, Genética y Evolución (IEGEBA), CONICET–Universidad de Buenos Aires, Buenos Aires, Argentina; 3 Instituto Nacional de Parasitología Dr. M. Fatala Chabén, Administración Nacional de Laboratorios e Institutos de Salud Dr. C.G. Malbrán, Buenos Aires, Argentina; 4 Laboratorio de Patologia e Biologia Molecular, Instituto Gonçalo Moniz/Fiocruz Bahia, Salvador, Brazil; 5 Paul G. Allen School for Global Health, Washington State University, Pullman, WA USA

**Keywords:** Vector-borne disease, Chagas disease, Heterogeneity, Parasite load, Infectiousness

## Abstract

**Background:**

The distribution of parasite load across hosts may modify the transmission dynamics of infectious diseases. Chagas disease is caused by a multi-host protozoan, *Trypanosoma cruzi*, but the association between host parasitemia and infectiousness to the vector has not been studied in sylvatic mammalian hosts. We quantified* T. cruzi* parasite load in sylvatic mammals, modeled the association of the parasite load with infectiousness to the vector and compared these results with previous ones for local domestic hosts.

**Methods:**

The bloodstream parasite load in each of 28 naturally infected sylvatic mammals from six species captured in northern Argentina was assessed by quantitative PCR, and its association with infectiousness to the triatomine *Triatoma infestans* was evaluated, as determined by natural or artificial xenodiagnosis. These results were compared with our previous results for 88 humans, 70 dogs and 13 cats, and the degree of parasite over-dispersion was quantified and non-linear models fitted to data on host infectiousness and bloodstream parasite load.

**Results:**

The parasite loads of *Didelphis albiventris* (white-eared opossum) and *Dasypus novemcinctus* (nine-banded armadillo) were directly and significantly associated with infectiousness of the host and were up to 190-fold higher than those in domestic hosts. Parasite load was aggregated across host species, as measured by the negative binomial parameter,* k*, and found to be substantially higher in white-eared opossums, cats, dogs and nine-banded armadillos (range: *k* = 0.3–0.5) than in humans (*k* = 5.1). The distribution of bloodstream parasite load closely followed the “80–20 rule” in every host species examined. However, the 20% of human hosts, domestic mammals or sylvatic mammals exhibiting the highest parasite load accounted for 49, 25 and 33% of the infected triatomines, respectively.

**Conclusions:**

Our results support the use of bloodstream parasite load as a proxy of reservoir host competence and individual transmissibility. The over-dispersed distribution of *T. cruzi* bloodstream load implies the existence of a fraction of highly infectious hosts that could be targeted to improve vector-borne transmission control efforts toward interruption transmission. Combined strategies that decrease the parasitemia and/or host–vector contact with these hosts would disproportionally contribute to *T. cruzi* transmission control.

**Graphical Abstract:**

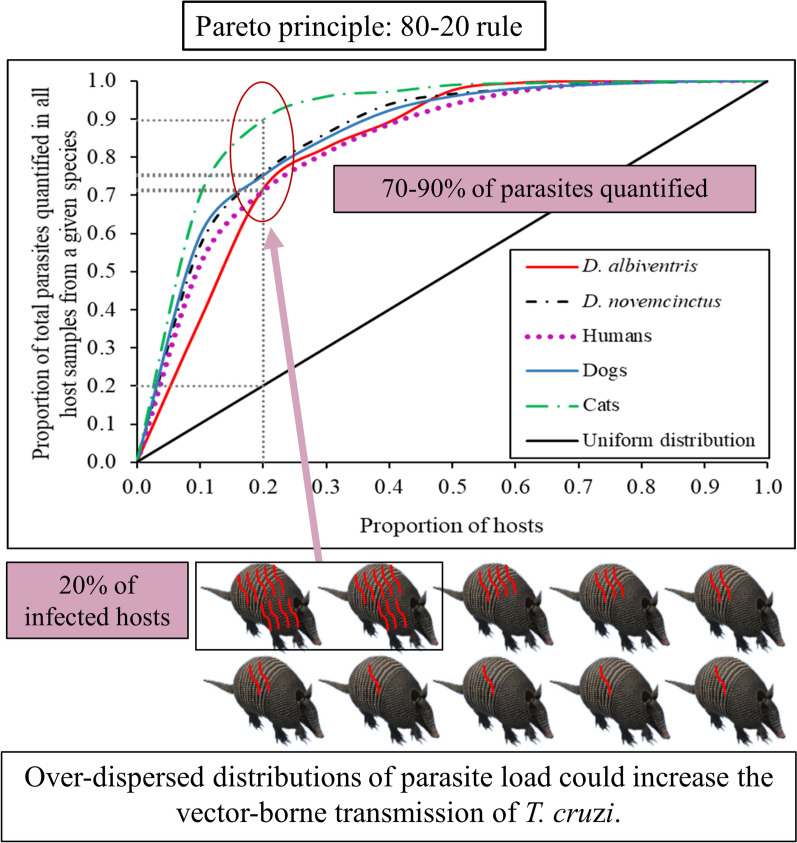

**Supplementary Information:**

The online version contains supplementary material available at 10.1186/s13071-022-05152-7.

## Background

The transmission of infectious diseases generally follows the Pareto principle or “80–20 rule,” namely that 80% of disease transmission is triggered by 20% of infected hosts [1, 2]. In vector-borne diseases, host infectiousness is the ability of an infected host to infect the vector. Infectiousness can be measured directly by xenodiagnosis or indirectly by host parasitemia. Consequently, an over-dispersed distribution of parasitemia (i.e. aggregated distribution of parasites) may modify transmission dynamics [3]. Mathematical modeling of host infectiousness in relation to parasitemia can provide further insights into this key relationship [4]. For multi-host parasite systems, it is particularly important to evaluate host competence, i.e. the ability of a host to acquire and transmit pathogens to other susceptible hosts or vectors [5], and to identify the (usually small) fraction of hosts that would disproportionally contribute to transmission. This fraction could be the target for optimized control strategies.

Chagas disease, one of the most important neglected tropical diseases in Latin America, is a vector-borne disease with a multi-host etiologic agent, *Trypanosoma cruzi* (Kinetoplastida, Trypanosomatidae) and several triatomine species that act as vectors. *Trypanosoma cruzi* is currently classified into seven discrete typing units (DTUs): TcI–TcVI and TcBat [6, 7]. All mammals are susceptible to *T. cruzi* infection, but dogs, cats, humans and synanthropic rodents play important roles as domestic reservoir hosts [8]. *Dasypus novemcinctus* (nine-banded armadillo) (Cingulata, Dasypodidae), *Didelphis albiventris* (white-eared opossum) (Didelphimorphia, Didelphidae) and several species of rodents are frequent sylvatic hosts of *T. cruzi* [9, 10]. The infectiousness of *T. cruzi*-infected hosts has been found to vary widely between and within host species, possibly implying that some species and individuals may contribute disproportionally to *T. cruzi* transmission [8, 9, 11–13].

Quantification of *T. cruzi* bloodstream load by quantitative (q) PCR in humans has shown heterogeneous levels of parasite concentration associated with age, DTU, stage of infection (i.e. chronic or acute), congenital infection and coinfection with other pathogens [14–21]. The parasite load of domestic animals and sylvatic hosts has been less frequently evaluated; wide variations have been reported and the tendency to be higher than in humans [11, 22–26]. Whether *T. cruzi* parasite load is aggregated or not and its degree of aggregation have not been addressed in domestic and sylvatic hosts, unlike in *Leishmania* and *Plasmodium* infections [27–29].

The Gran Chaco region is the distribution center of the main domestic vector of Chagas disease, *Triatoma infestans* (Hemiptera, Triatominae). As part of a broader research program on the eco-epidemiology and control of this infectious disease in the Argentine Chaco [30], we assessed *T. cruzi* infection in humans, dogs, cats and several sylvatic mammalian species by means of serodiagnosis, xenodiagnosis and PCR (both conventional and qPCR). *Trypanosoma cruzi* TcV and TcVI were the most prevalent DTUs found in domestic hosts [31, 32], while *T. cruzi* TcI and TcIII were the only DTUs found in white-eared opossums and various species of armadillos, respectively [13], implying separated transmission cycles. The mean infectiousness to the vector was higher in *D. novemcinctus* (74%) and *D. albiventris* (56%), followed by dogs (48%) and cats (44%), and was only 5% in humans. Median parasite load estimated by qPCR displayed a similar trend as infectiousness for domestic hosts [11–13]. However, the parasite load of sylvatic hosts has not yet been quantified in the Gran Chaco region.

In this study, we assessed the bloodstream parasite load of six sylvatic host species infected with *T. cruzi*, namely *D. novemcinctus*,* D. albiventris*,* Euphractus sexcinctus* (six-banded armadillo) (Cingulata, Dasypodidae), *Tolypeutes matacus* (southern three-banded armadillo) (Cingulata, Dasypodidae), *Chaetophractus vellerosus* (screaming hairy armadillo) (Cingulata, Dasypodidae) and *Conepatus chinga* (Molina's hog-nosed skunk) (Carnivora, Mephitidae), from two eco-regions of northern Argentina and evaluated its association with infectiousness to *T. infestans*. We compared these results with previous ones for humans (*n* = 88), dogs (*n* = 70) and cats (*n* = 13) from rural areas of Pampa del Indio, Chaco, quantified the degree of parasite over-dispersion and modeled the relationship between host infectiousness and parasite load. We hypothesized that: (i) the bloodstream parasite load of both sylvatic mammals (i.e. *D. novemcinctus*,* D. albiventris*) would be directly associated with host infectiousness; (ii) the bloodstream parasite loads of both sylvatic mammals would be higher than that of humans and domestic animals; and (iii) the parasite load would be highly aggregated across host species and fit the Pareto principle.

## Methods

### Study area

Samples were mainly collected in the municipality of Pampa del Indio (26°2′0″S, 59°55′0″W), Chaco Province, Argentina, which is located in the transition zone between the dry and humid Chaco. The rural area of the municipality was divided in four sections (areas I–IV); in all four sections, a similar intervention protocol to suppress the vector-borne transmission of *T. cruzi* had been scaled up since 2007. *Triatoma infestans* house infestation prior to a community-wide residual spraying with pyrethroid insecticide ranged from 21% to 46% [33–35]. Sustained control actions led to a substantial reduction of *T. cruzi* infection in the human and dog populations that was compatible with the interruption of domestic vector-borne transmission to humans [30]. In order to increase the sample size of sylvatic hosts, we additionally included four *D. albiventris* samples from the Southern Cone Mesopotamian savannah eco-region. These samples were collected in the Capital (27°24′S, 55°55′W) and Candelaria (27°22′S, 55°34′W) Departments of Misiones Province, Argentina [36], which had been certified to be free of *T. infestans-*mediated transmission of *T. cruzi* in 2011 [37].

### Study design

This study profits from previous cross-sectional studies in which *T. cruzi* infection was assessed in sylvatic and domestic mammals by serological, parasitological and/or molecular tests between 2008 and 2017 [11–13, 22, 36, 38]. Humans, dogs and cats were considered to be seropositive to *T. cruzi* if shown to be reactive by at least two different serological tests [39]. Dogs and cats were also examined by xenodiagnosis; they were considered to be infected with *T. cruzi* if seropositive or xenodiagnosis-positive [40]. Sylvatic mammals were considered to be infected if xenodiagnosis-positive or if kinetoplast DNA-PCR (kDNA-PCR) results (based on guanidine–EDTA blood samples [GEB]). For further confirmation, kDNA-PCR-positive GEB samples from sylvatic hosts that were xenodiagnosis-negative were subsequently tested by satellite DNA-PCR (SAT DNA-PCR) or the rectal contents of triatomines used in the xenodiagnosis were tested by kDNA-PCR [13, 36]. In this study, we quantified the bloodstream parasite loads of infected sylvatic host species and compared these with previous estimates of parasite loads and infectiousness of *T. cruzi*-seropositive dogs, cats and humans [11, 12, 22]. A thorough description of the origin of the samples (including host species, prevalence of infection, infectiousness, parasite load and parasite DTU) is given in Additional file 1: Table S1 and Additional file 2: Dataset S1.

### Xenodiagnosis and host infectiousness

Artificial xenodiagnosis tests were performed on *T. cruzi*-seropositive humans. For each test, 20 fourth-instar nymphs of laboratory-reared *T. infestans* (kept unfed for at least 3 weeks) were fed 3 ml of heparinized blood from each patient using a blood-feeding device. Plastic centrifuge tubes (50 ml) with their ends cut were used for collecting heparinized blood. A latex membrane was attached to the tubes by means of a rubber O-ring; a copper tube was coiled around the centrifuge tubes containing the blood to maintain it at 37 °C; and hot water running inside the tube was pumped by means of an electric motor [12]. The elapsed time between blood extraction and the onset of feeding was < 5 min. Natural xenodiagnosis in dogs and cats was performed with 10 or 20 uninfected, laboratory-reared fourth-instar nymphs of *T. infestans* exposed to each animal’s belly for 20 min. Anaesthetized sylvatic animals were examined by natural xenodiagnosis using 5–20 uninfected fourth-instar nymphs of *T. infestans* (depending on body size) applied onto the belly of the host for 25 min [13]. All humans and animals tested by xenodiagnosis were subsequently re-exposed for 10 min if following the initial exposure most insects had not blood-fed to repletion as evaluated by the naked eye. Each triatomine was individually examined for *T. cruzi* infection by optical microscopy (OM) at ×400 magnification at 30 and 60 days after exposure. Triatomine molting rates averaged 11% for humans, 12% for dogs, 8% for cats and 14% for sylvatic mammals. The infectiousness to the vector was calculated as the total number of triatomines infected with *T. cruzi* divided by the total number of triatomines exposed to the infected host and examined for infection at least once. Infectiousness results from sylvatic mammals included in this study have been published elsewhere [13, 36, 38].

### Molecular analysis

All GEB samples were heated in boiling water. Prior to DNA extraction, an internal amplification control DNA (IAC) was added to a 400-μl GEB aliquot and DNA was extracted as previously reported [11]. Purified DNA was eluted in 200 μl of distilled water and used as template for qPCR amplification. Bloodstream parasite load was quantified by amplifying a *T. cruzi* satellite DNA with a standard curve reflecting a dynamic range of *T. cruzi* between 0.1 and 10^6^ parasites/ml, as described elsewhere [14]. DNA quantification was normalized according to the identified DTU [41]. Parasite load of 66 humans without DTU identification was normalized as if they were infected with TcV given that this DTU predominates in human samples from the Argentinean Chaco [32, 42–44]. We expressed parasite DNA concentration as equivalent amounts of parasite DNA per ml (Pe/ml). The cut-off value of this method is 0.14 Pe/ml [14]. Samples in which parasite DNA was not detected by qPCR were considered to have zero parasite load. Twenty-six samples (22 humans, 3 dogs, 1 cat) fell within the detectable but non-quantifiable range of the dynamic curve (i.e. > 0 and < 0.14 Pe/ml).

### Host infectiousness model

Host infectiousness of domestic and sylvatic mammals was estimated using a mechanistic non-linear model described by Miller et al. [14] (i.e. Model 1). This model considers that the probability of a vector becoming infected in a single feeding event depends on host parasitemia, vector blood meal size, the minimum number of parasites required to infect the vector and the probability of becoming infected related to intrinsic factors, such as incompatibility with vector gut microbiota. The model has two parameters: B1, the probability that a vector acquires the infection, and B2, the probability that a host harboring a parasite load of one parasite per milliliter infects a vector. Blood meal size (*υ*) and the minimum number of parasites necessary to infect a vector (*θ*) are components of B2. In the case of *T. cruzi*, both *υ* and *θ* are related to vector species and stage. We fitted the data to Model 1 and to a second model without B1 (Model 2).

Model 1 is expressed as:

$${\text{Infec}}\left( x \right) = B{1}*({1} - \left( {{\text{exp}}\left( { - x*B{2}} \right)} \right)$$

where *x* represents parasitemia.

Alternatively, a logistic model was fitted to the data (Model 3). This model also has two parameters (obtained from the logistic curve graph [45]) that differ from the coefficients B1 and B2 used in model 1 as is expressed as:

Model 3

$${\text{Infec}}\left( x \right) = {1 \mathord{\left/ {\vphantom {1 {\left( {{{{1} + {\text{exp}}\left( x{{\text{mid}} - x} \right)} \mathord{\left/ {\vphantom {{{1} + {\text{exp}}\left( {{\text{xmid}} - x} \right)} {{\text{scal}}}}} \right. \kern-\nulldelimiterspace} {{\text{scal}}}}} \right)}}} \right. \kern-\nulldelimiterspace} {\left( {{{{1} + {\text{exp}}\left( {{\text{xmid}} - x} \right)} \mathord{\left/ {\vphantom {{{1} + {\text{exp}}\left( {{{xmid}} - x} \right)} {{\text{scal}}}}} \right. \kern-\nulldelimiterspace} {{\text{scal}}}}} \right)}}$$

where* x* represents parasitemia,* xmid* is the inflection point on the curve and* scal* is a growth scale. The initial value for the* xmid* parameter is the parasite load at which half of the maximum observed infectiousness is reached. The initial value for the* scal* parameter comes from the difference between the parasite load corresponding to an infectiousness of 0.75 (i.e. 75% of the asymptote, which is equal to 1, the maximum proportion of infected triatomines) and that corresponding to the* xmid* [45].

### Data analysis

Wilson binomial 95% confidence intervals (CIs) were used for proportions. Given that skewed parasite distributions among hosts are usually observed, we calculated the median bloodstream parasite load and its confidence interval [46]. We compared the median parasite loads of sylvatic and domestic host species by means of a Moods median test [46]. This test does not allow samples with an amount equal to zero; therefore, we added 0.01 Pe/ml to each of these samples. We also compared the medians of bloodstream parasite load by sex and stage within each host species by Kruskal–Wallis non-parametric tests. The probability used for nominal statistical significance was 5%. *Didelphis albiventris* opossums were assigned to life-stage class (juvenile, pre-adult or adult) based on tooth eruption [13]. *Didelphis albiventris* samples from Misiones and Chaco provinces were pooled for analysis.

The relationship between infectiousness to the vector of *T. cruzi*-infected *D. albiventris*, *D. novemcinctus*, dogs, cats and humans and bloodstream parasite load was analyzed using generalized linear models (GLMs) implemented in the R environment (version 4.0.2; 2020; R Foundation for Statistical Computing, Vienna, Austria) with the packages MuMIn [47], ResourceSelection [48] and car [49]. Infectiousness was the response variable, and a binomial distribution with logit link function was considered. We assessed whether the dependent variable was associated with bloodstream parasite load (a continuous variable, in Pe/ml) and host species (with humans as the reference level) and adjusted it by the triatomine molting rate on individual hosts as a proxy of blood intake. Multicollinearity was evaluated by the variance inflation factor (VIF), and interaction terms were added and dropped from the final model if found not to be significant at a nominal significance level of 5%.

To assess whether bloodstream parasite load was associated with host species and sex we performed a negative binomial regression. The dependent variable was a continuous variable (in Pe/ml). The independent variables were host species (five levels: 1 = humans; 2 = dogs; 3 = cats; 4 = *D. albiventris*; 5 = *D. novemcinctus*) and sex (0 = female; 1 = male). DTU was excluded from the regression model due to multicollinearity, as determined by the VIF. Interaction terms were added and dropped from the final model if found to be not significant at a nominal significance level of 5%. The likelihood-ratio test (LR) was used to assess over-dispersion by the alpha parameter of dispersion from the Poisson distribution (i.e. alpha = 0 means Poisson regression presents a better fit to the data).

To evaluate the degree of aggregation of bloodstream parasite load, we estimated the values of the parameter of dispersion “*k*” from the negative binomial distribution using maximum likelihood procedures [46]; we expected *k*  ≤ 5 in the case of aggregation [50]. We used the Pareto fraction to evaluate the heterogeneous distribution of parasite load and its relationship with the total number of *T. cruzi*-infected *T. infestans* obtained by xenodiagnosis by host species. The Pareto fraction is defined as the proportion of the total hosts (by species) for which it is possible to say that a proportion *X* with the highest bloodstream parasite load accounted for a proportion 1 − *X* of the total bloodstream parasite load and of *T. cruzi*-infected *T. infestans*. We excluded from analysis a *D. novemcinctus* with an extreme parasite load (8325 Pe/ml) because it was an outlier data as determined by the distribution of studentized residuals. If this sample were to be included in the aggregation analysis, the* k* index would drop to 0.25 (i.e. a greater degree of aggregation) and the 10% of *D. novemcinctus* samples with the highest parasite load**s** would harbor 85% of the quantified parasites.

The infectiousness models were fitted by non-linear regression with mixed effects [45] implemented in the R environment (version 4.0.2; 2020; R Foundation for Statistical Computing, Vienna, Austria) using the package “nlme” for domestic hosts. Because of the low number of sylvatic hosts, we only included *D. albiventris* opossums and *D. novemcinctus* armadillos and fitted the models using the “nls2” package without mixed effects. Three models (Models 1–3) and two parasite load datasets were used (one dataset for dogs, cats and humans, and one dataset for *D. novemcinctus* and *D. albiventris*). For the domestic hosts’ dataset, we included the “varConstPower” variance function from the “nlme” library in the models and host age (in years) as a stratification variable for the estimation of variance parameters. We used the Akaike’s information criterion (AIC) for model selection [51]. All confidence intervals were estimated using a hierarchical bootstrapping method by resampling with replacement both at the level of the group (i.e. the random variable which was the type of host) and within groups; in this level, the method samples the residuals and adds them back to the predictions [52].

## Results

### Bloodstream parasite load in sylvatic hosts

Of the 28 sylvatic host blood samples, 23 (82%) fell within the quantifiable range of parasite load (i.e. ≥ 0.14 Pe/ml); no parasite DNA was detectable in the remaining five samples. All qPCR-negative mammals were from Pampa del Indio: three *D. albiventris* and one *E. sexcinctus* were negative by xenodiagnosis and kDNA-PCR-positive, and one *T. matacus* armadillo was xenodiagnosis-positive and kDNA-PCR-negative. The median parasite load did not differ significantly between the nine *D. albiventris* opossums from Pampa del Indio (10.7 Pe/ml; 95% CI: 0.1–61.1) and the four opossums captured in Misiones (median = 52.5 Pe/ml; Moods median test, two-sided *P* = 0.55). The median parasite load of all *D. albiventris* opossums (13.8 Pe/ml; 95% CI: 0.1–61.1) was not significantly different from that of *D. novemcinctus* armadillos (38.3 Pe/ml; 95% CI 4.1–269.1; Moods median test, two-sided *P* = 0.39). The parasite loads of the only *T. cruzi*-infected specimens of *E. sexcinctus*,* C. vellerosus* and *C. chinga* were 4.2, 40.0 and 7.0 Pe/ml, respectively. In *D. albiventris* opossums, the median parasite load did not differ significantly between life-cylce stages (Kruskal–Wallis H-test, *H* = 2.04, *df* = 1, *P* = 0.18) or sexes (Kruskal–Wallis H-test, *H* = 0.46, *df* = 1, *P* = 0.52). Median parasite load was lower in female armadillos (38.3 Pe/ml) than in males (108.3 Pe/ml) but the difference was not significant (Kruskal–Wallis H-test, *H* = 0.53, *df* = 1, *P* = 0.55).

**Fig. 1:**
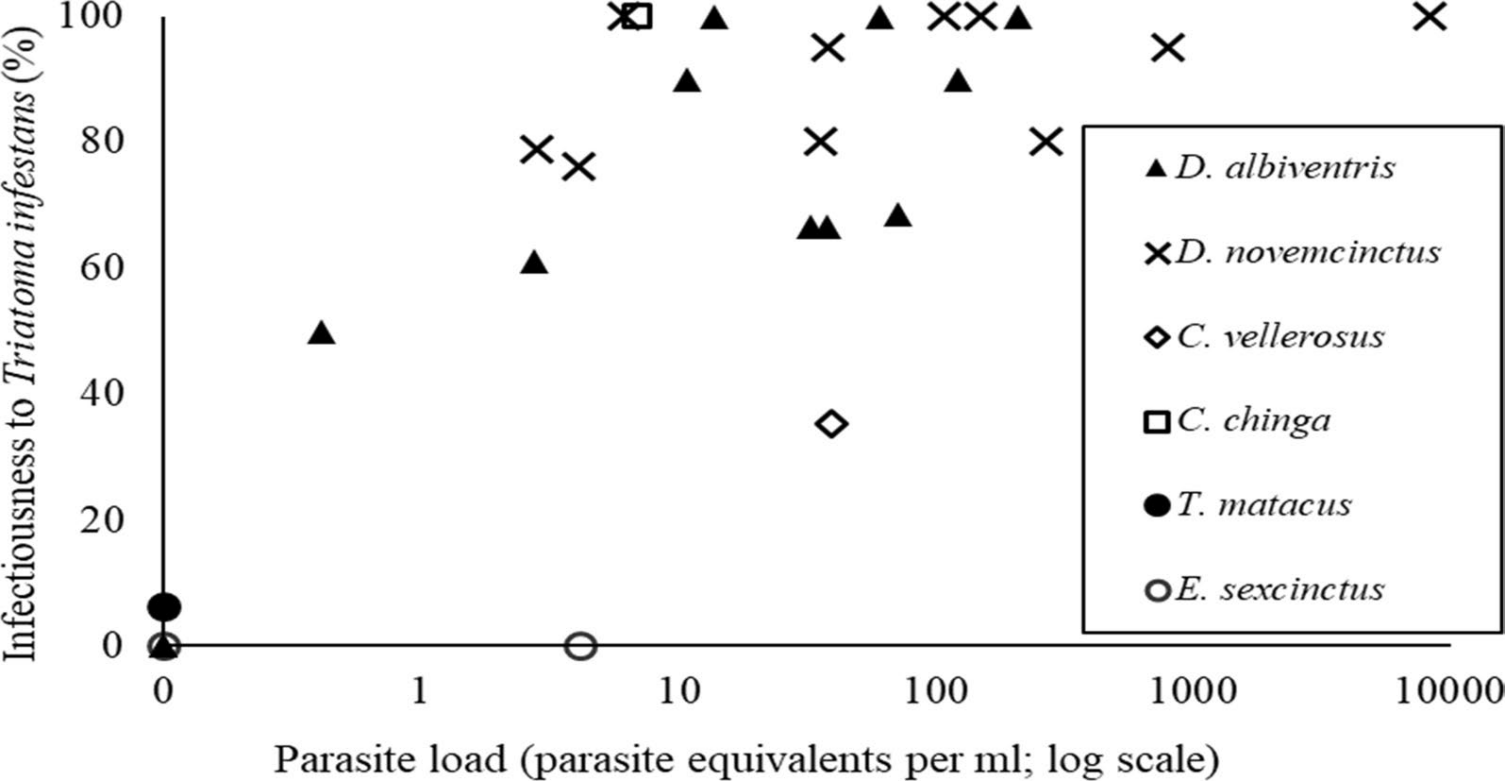
Distribution of infectiousness of *Trypanosoma cruzi*-infected sylvatic hosts to the vector *Triatoma infestans*, at Pampa del Indio 2008–2010 and Misiones 2011

The infectiousness of infected *D. albiventris* and *D. novemcinctus* steeply increased with bloodstream parasite load, reaching 100% between 6 and 13 Pe/ml (Fig. 1). Infectiousness was positively associated with bloodstream parasite load (odds ratio [OR]: 1.02; 95% CI: 1.01–1.03; *P* = 0.03) and host species. *Dasypus novemcinctus* exhibited the highest odds ratio (OR: 60.73; 95% CI: 6.87–1501.36: *P* < 0.01), followed by *D. albiventris* (OR: 12.95; 95% CI: 2.32–78.46; *P* < 0.01), dogs (OR: 12.64; 95% CI: 4.58–43.03; *P* < 0.01) and cats (OR: 9.50; 95% CI: 1.82–49.73; *P* < 0.01) (Fig. 3). Infectiousness was not significantly associated with triatomine molting rate (OR: 2.83; 95% CI: 0.14–69.83; *P* = 0.50).

### Parasite load and infectiousness in domestic and sylvatic hosts

The median bloodstream parasite load of *D. albiventris* and *D. novemcinctus* was 1.4- to 190-fold higher than that of cats (9.7 Pe/ml; 95% CI: 1.6–96.9), dogs (5.5 Pe/ml; 95% CI: 2.6–13.0) and humans (0.2 Pe/ml; 95% CI: 0.1–0.3). The negative binomial regression model was found to fit the data better than the Poisson model when the model for bloodstream parasite load included host species and sex as independent variables (alpha = 2.2; 95% CI: 1.8–2.7; *P* < 0.01). Bloodstream parasite load was significantly associated with host species but not with sex (*P* = 0.59) (LR: 238.3; *P* < 0.01) (Table 1).

**Table 1:** *Trypanosoma cruzi* parasite load in domestic and sylvatic hosts, at Pampa del Indio 2008–2017 and Misiones 2011

Predictor	Number of hosts^a^	Median parasite load (Pe/ml)	incidence rate ratio	Standard error	95% Confidence interval
Host species					
Humans	88	0.2	1.0	–	–
*Didelphis albiventris*	13	13.8	97.8	46.1	38.9–246.2*
*Dasypus novemcinctus*	10	38.3	2354.3	1262.7	822.9–6735.7*
*Canis lupus familiaris*	68	5.5	49.4	14.6	27.7–88.1*
*Felis catus*	12	9.7	279.5	138.7	105.7–739.3*
Sex					
Female	79	0.6	1.0	–	–
Male	107	1.4	1.2	0.3	0.7–2.0

*Statistically significant

^a^Two dogs and 1 cat for which sex was not determined were excluded from this analysis

**Fig. 2:**
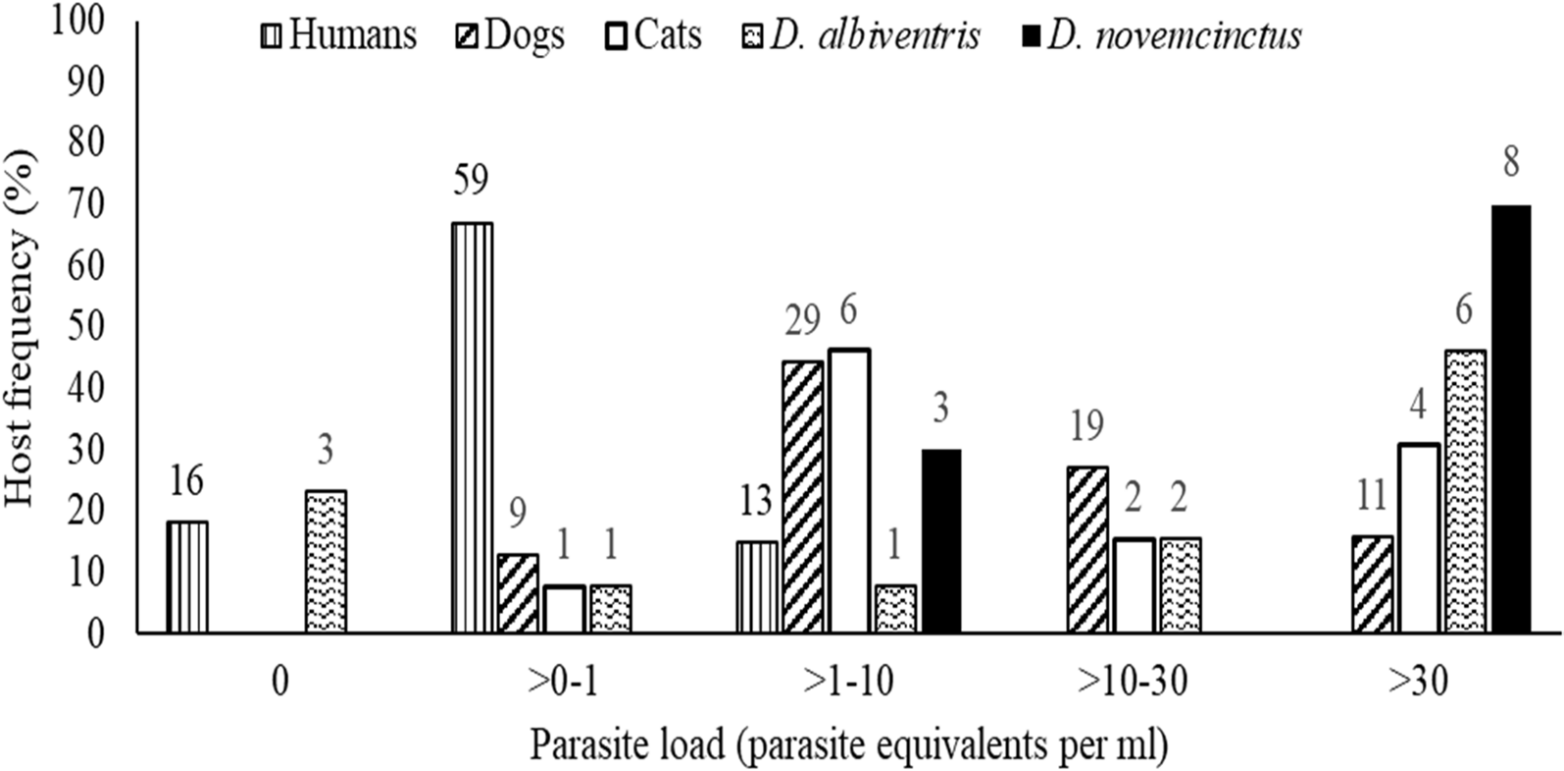
Frequency distribution of parasite load of *T. cruzi*-infected domestic and sylvatic hosts, at Pampa del Indio 2008–2017 and Misiones 2011. The number of hosts is indicated above each bar

The frequency distribution of parasite load was highly skewed across all host species with a unimodal pattern for each species. The majority of armadillos and white-eared opossums exhibited high parasite loads; for example, 70% of *D. novemcinctus* and 46% of *D. albiventris* exhibited a bloodstream parasite load ≥ 30.0 Pe/ml (Fig. 2). In contrast, most humans exhibited low parasite loads whereas most dogs and cats had intermediate ones. The bloodstream parasite load of most seropositive humans (85%) was < 1 Pe/ml, with parasitaemia undetectable by qPCR in 18% of these. Nearly half of the study dogs (44%) and cats (46%) had parasite loads between 1 and 10 Pe/ml (Fig. 2). Parasite aggregation measured by the negative binomial parameter *k* was higher in cats and white-eared opossums (*k* = 0.3) and in nine-banded armadillos and dogs (*k* = 0.4 and 0.5, respectively) than in humans (*k* = 5.1) (Table 2).

**Table 2:** Degree of parasite aggregation measured by the negative binomial dispersion index, *k*

Host species	Number of hosts	Parasite load (Pe/ml)		*k*
		Mean	Variance	
*Didelphis albiventris*	13	43.3	3825.3	0.3
*Dasypus novemcinctus*	9^a^	157.3	66,050.9	0.4
Dogs	70	22.9	2612.7	0.5
Cats	13	121.6	90,505.9	0.3
Humans	88	0.4	0.6	5.1

^a^One *D. novemcinctus* with 8325 parasites/ml was considered to be an outlier and excluded from analysis

**Fig. 3:**
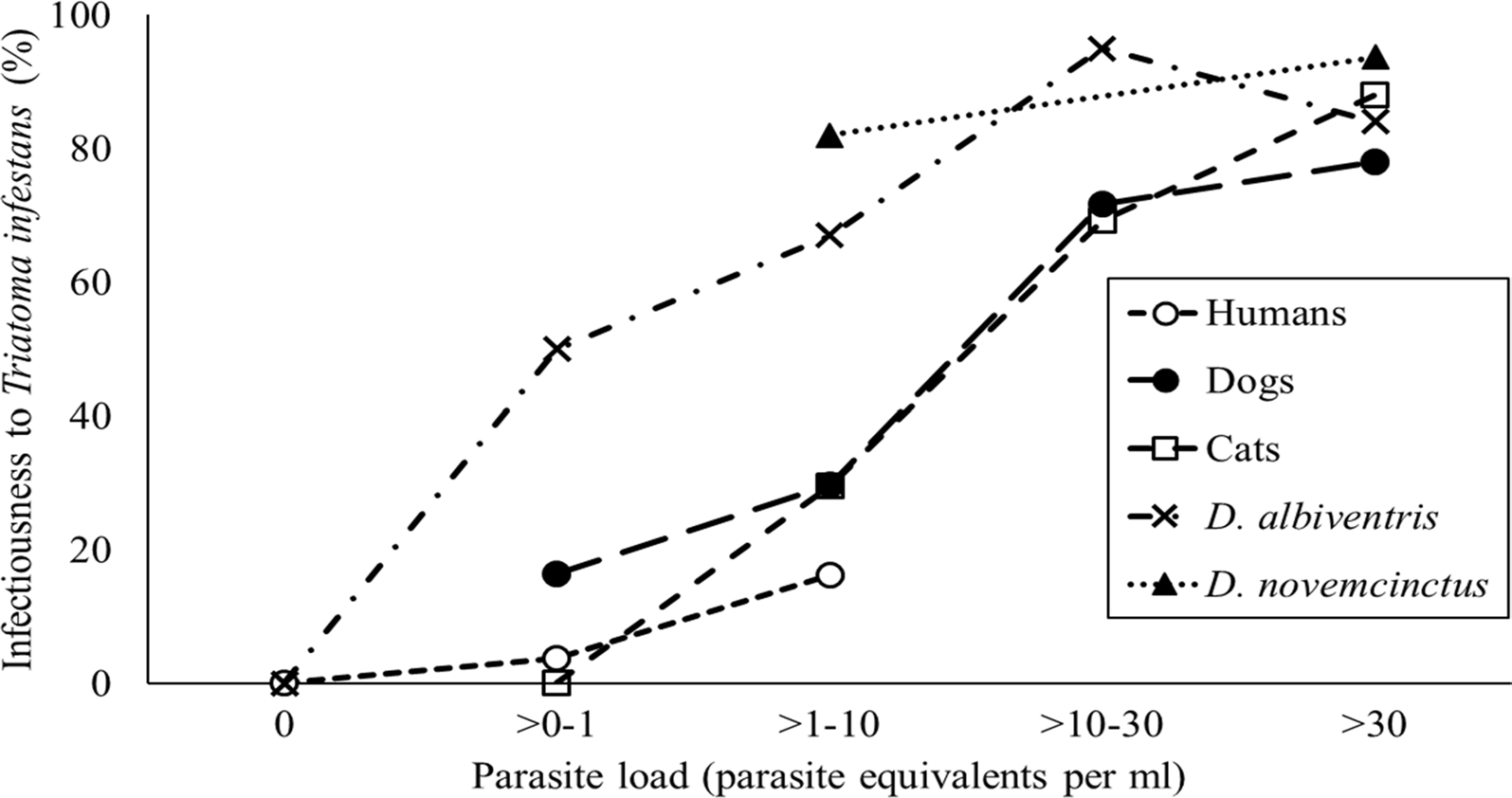
Host infectiousness to *T. infestans* according to parasite load in *T. cruzi*–infected domestic and sylvatic mammals, at Pampa del Indio 2008–2017 and Misiones 2011

*Didelphis albiventris* and *D. novemcinctus* were more infectious to the vector than any domestic host species (i.e. humans, dogs and cats) across the range of parasite loads observed (Fig. 3). The greatest difference between domestic and sylvatic hosts was between 1 and 10 Pe/ml. Dogs, cats, white-eared opossums and nine-banded armadillos were similarly infectious for ≥ 30 Pe/ml (Fig. 3).

### Pareto analysis

**Fig. 4:**
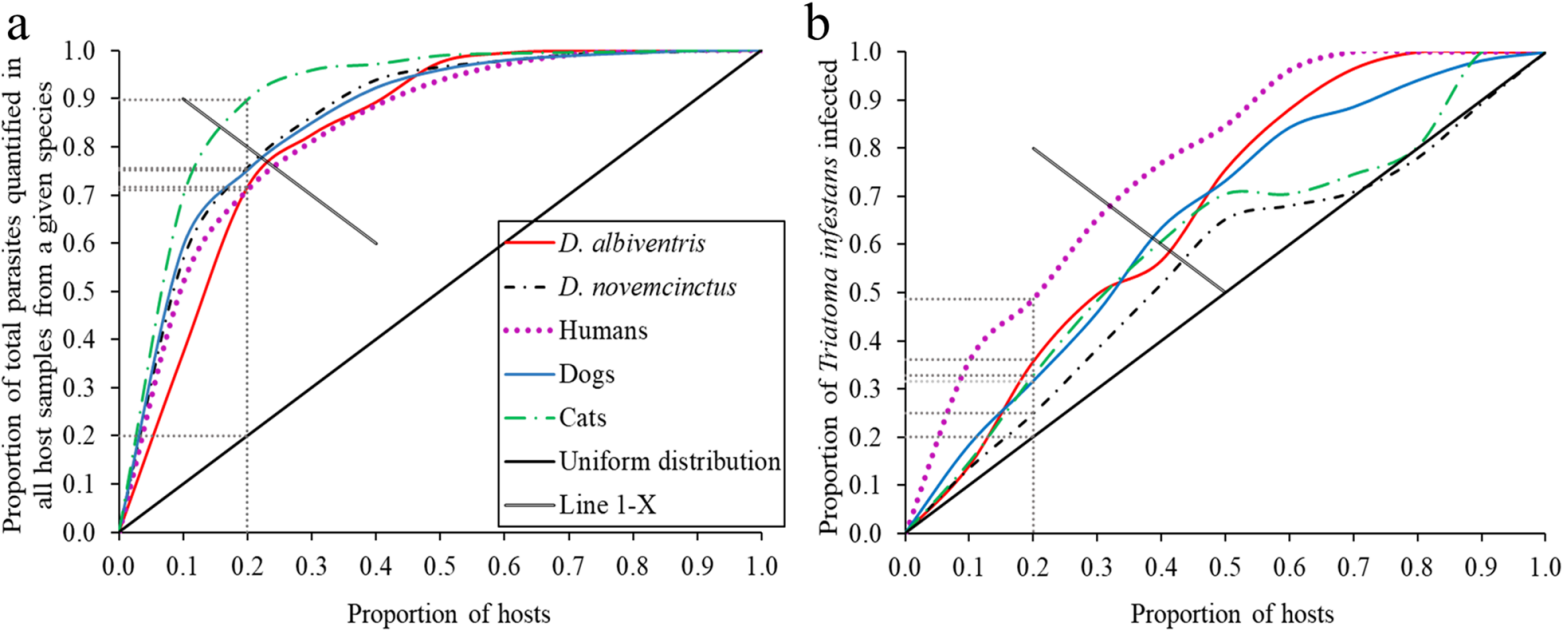
Pareto principle for *T. cruzi* parasite load (**a**) and for *T. cruzi*-infected *T. infestans* in xenodiagnostic tests (**b**) of domestic and sylvatic host species, at Pampa del Indio 2008–2017 and Misiones 2011

The bloodstream parasite load was aggregated across host species and found to closely fit the Pareto principle, with 20% of host samples with the highest parasite loads in each species harboring 70–90% of parasite equivalents measured by qPCR in 1 ml of blood (Fig. 4a). In Fig. 4, the point at which the distribution line of each host species crosses the line 1 − *X* (grey line) is the Pareto fraction for each host species. The Pareto fraction for parasite load distribution across species was approximately 75–24 for *D. albiventris*, 80–20 for *D. novemcinctus*, 78–22 for humans and dogs and 84–16 for cats. The Pareto fraction for *T. cruzi*-infected*T. infestans* across species was very distant from the “80–20 rule,“ with the 20% of humans exhibiting the highest parasite load accounting for 49% of the infected bugs. Similarly, the 20% of domestic or sylvatic mammals displaying the highest parasite load was responsible for 25–33% of the infected bugs (Fig. 4b).

### Infectiousness model

**Fig. 5:**
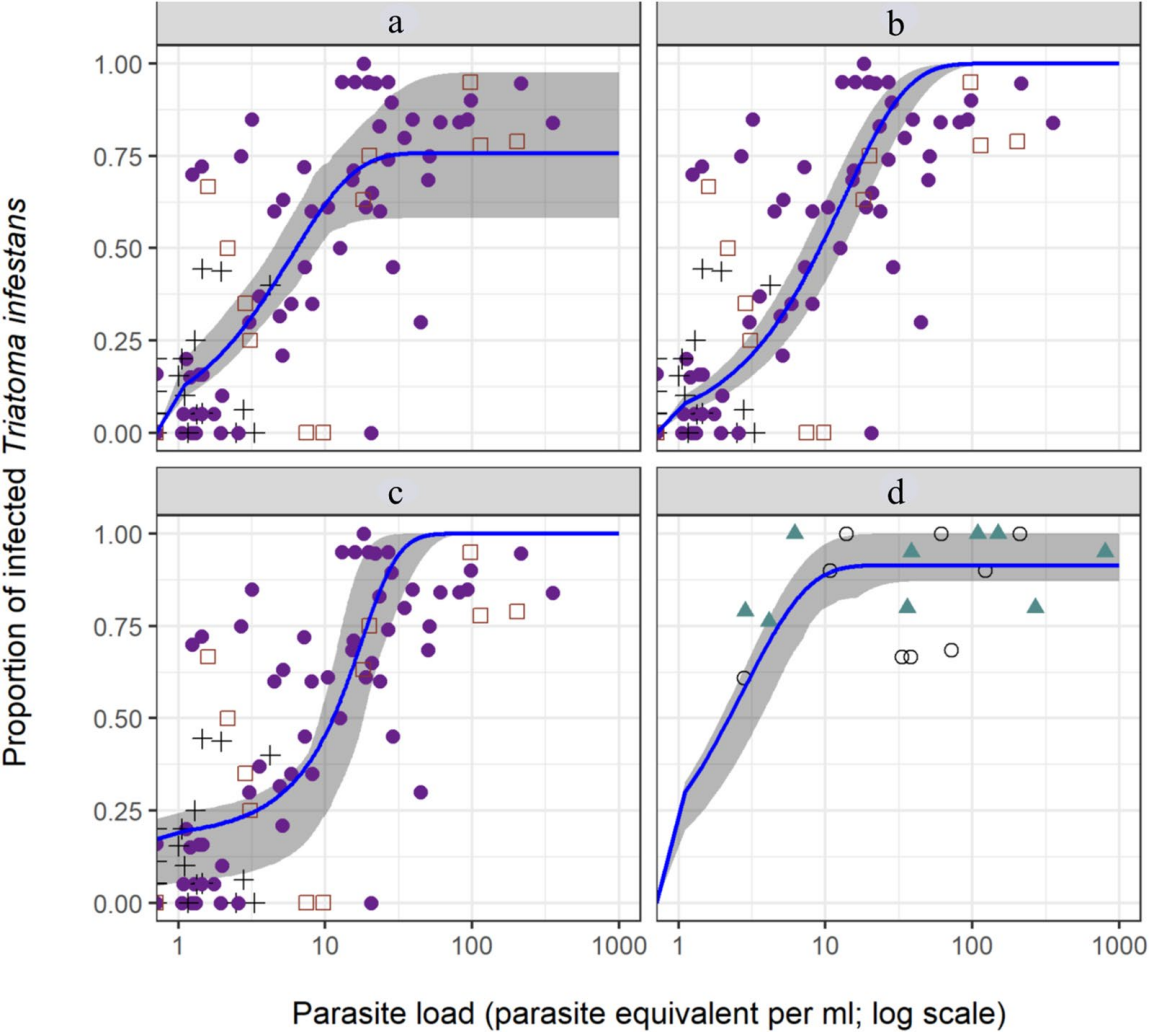
Observed and predicted host infectiousness, with 95% confidence intervals (shaded areas around solid blue line), according to bloodstream parasite loads in *T. cruzi*-infected domestic and sylvatic mammals, at Pampa del Indio 2008–2017 and Misiones 2011. Hosts with zero parasite load were pooled with those exhibiting 0.01 Pe/ml.** a** Model 1,** b** Model 2,** c** logistic model for domestic animals,** d** Model 1 for sylvatic hosts. Symbols:  + represents humans; open square, cats; filled circle, dogs; filled triangle,* Dasypus novemcinctus* (9-banded armadillo); open circle, *Didelphis albiventris* (white-eared opossum)

Model 1 exhibited the lowest AIC values for both datasets followed by Model 2 (Table 3; Fig. 5a–d). The main difference between Models 1 and 2 in domestic hosts was observed for parasite loads > 10 Pe/ml, in which infectiousness reached an asymptote at 80% and 100%, respectively, and where a wide confidence interval was observed for Model 1 (Fig. 5a, b). In Model 1, a similar value for the B1 parameter was determined for both host datasets; however, the parameter B2 was twice as high for the sylvatic hosts’ model than for that of the domestic hosts (Table 3). The logistic model had the highest AIC value (Fig. 5c) and exhibited a poor fit to the data, especially for low parasite loads, in which the predicted infectiousness was never < 20%, unlike in the observed data.

**Table 3:** Estimated parameters for three non-linear models of host infectiousness for domestic and sylvatic mammals infected with *Trypanosoma cruzi*

Model	No. of hosts	Model parameters (standard error)					
		*df*	B1^a^	B2^b^	Xmid^c^	scal^c^	AIC
Domestic hosts							
1	171	167	0.76 (0.04)	0.17 (0.03)	NA	NA	− 94.69
2	171	168	NA^d^	0.10 (0.01)	NA	NA	− 70.17
Logistic	171	167	NA	NA	11.30 (1.14)	7.19 (1.88)	− 66.69
Sylvatic hosts							
1	23	20	0.91 (0.04)	0.36 (0.10)	NA	NA	− 16.53
2	23	21	NA	0.48 (0.12)	NA	NA	− 14.34
Logistic	23	21	NA	NA	10.00 (7.07)	22.00 (10.86)	9.63

*AIC* Akaike’s information criterion

^a^ B1 is the probability that a vector becomes infected

^b^ B2 is the probability that a host with a parasite load of one parasite per milliliter (1 Pe/ml) infects a vector

^c^ In the logistic model, xmid is the inflection point on the curve and scal is a growth scale

^d^NA: Does not apply. The parameter was not included in the model

## Discussion

We quantified the bloodstream load of *T. cruzi* from six sylvatic host species from northeastern Argentina and compared these results with the parasite load determined for local domestic hosts. This study may be the first attempt of this kind for two of the main sylvatic reservoir hosts of *T. cruzi*, *D. albiventris* opossums and *D. novemcinctus* armadillos. All examined sylvatic and domestic host species exhibited an aggregated parasite load as determined by the negative binomial distribution; the parasite load distribution followed the Pareto principle. Over-dispersed distributions of parasite load associated with heterogeneous host infectiousness have also been reported for other vector-borne diseases and shown to influence transmission dynamics [4, 27, 53–56].

The “80–20 rule” described by Woolhouse et al. [2] suggests that heterogeneities in vector-host contact rates can increase the basic reproductive number *R*_0_. If transmission can be directly linked to the intensity of parasitemia, the small fraction of hosts with the highest parasite load would disproportionally contribute to transmission [4, 28]. In our study, the distribution of host infectiousness to the vector deviated from the Pareto principle. The 20% of hosts with the highest parasite load were responsible for 49% and nearly 30% of triatomine infections derived from *T. cruzi*-infected humans and domestic or sylvatic mammals, respectively. These percentages of infected triatomines were lower than expected; such deviations may be explained by the details of the xenodiagnosis protocol, which sought to maximize the likelihood of vector infection via exposure times. Thus, host infectiousness in dogs, cats and sylvatic mammals was skewed toward high values, with few individuals having zero infectiousness. Although we did not address vector–host contact rates specifically in this study, the small fraction of *T. cruzi*-infected hosts exhibiting the highest parasite loads could contribute disproportionately to parasite transmission, as previously suggested for domestic dogs and cats infected by *T. cruzi* [57].

The few published studies that have evaluated *T. cruzi* parasitemia in sylvatic hosts mainly focused on synanthropic and wild rodent species from Chile and Mexico, which showed a median parasite load between 1.0 and 6.2 Pe/ml [23, 24, 26, 58] In one study, *T. cruzi-*infected marsupials, such as *Didelphis marsupialis* (Didelphimorphia, Didelphidae) and *Marmosa murina* (Didelphimorphia, Didelphidae), from Colombia were qPCR-positive, with a median parasite load of 27.4 and 0.2 Pe/ml, respectively [25]. In our study, the parasite load of *D. albiventris* from two regions of northern Argentina that differed in the intensity of *T. cruzi* transmission was not significantly different. The overall median parasite load for both *D. albiventris* populations did not differ from that determined for *D. marsupialis* in Colombia, and was nearly fourfold higher than the parasite load for rodents in Chile and Mexico. The parasite load of *D. novemcinctus* and *C. vellerosus* did not differ significantly from that recorded for *D. albiventris* in this study. The few *T. matacus* and *E. sexcinctus* examined showed a low parasite load, similar to that in rodents, with low or zero infectiousness to the vector. An increasing trend of bloodstream parasite concentration from rodent species to *Didelphis* sp. and *D. novemcinctus* is apparent among all the above-mentioned sylvatic host species. This trend agrees with the high reservoir host competence reported for opossums and *D. novemcinctus* [10, 13, 59].

Immunologically competent humans in the chronic phase of *T. cruzi* infection have been found to display variable parasite loads, with a median range of 0.1–2.3 Pe/ml across South America [15–17, 60–62]. This range is similar to the parasite load found in chronic humans from Pampa del Indio. The median parasite load for *D. albiventris* and *D. novemcinctus* was up to 190-fold higher than that for humans in Pampa del Indio and elsewhere, and up to sevenfold higher than that of dogs and cats. A much lower median parasite load of 0.1 Pe/ml was reported in goats from Mendoza Province, Argentina [63]. This heterogeneous distribution of parasite load across sylvatic and domestic host species suggests a wide variability in parasite–host interactions, and is likely determined by multiple factors. First, (co-)infections, biotic and abiotic conditions and anthropogenic factors can affect the host immune system and contribute to the heterogeneous distribution of parasite load [64]. Secondly, a long-standing host–parasite association, in which the marsupial genus *Didelphis* and placental mammals, such as armadillos, have been proposed as the ancestral hosts of *T. cruzi* [10, 65]. Co-evolution could also be associated with parasite genotype selection [9, 10, 65–67]. The almost exclusive association between sylvatic host species and parasite DTUs precluded us from analyzing the potential effect of parasite genotypes on parasitemia. Experimental studies with animal models and observations in humans suggest that the diversity of parasite genotypes may in part account for the variability in parasite load [16, 17, 19, 59]. However, earlier studies found no significant differences in the median parasite loads of humans, dogs and cats with different DTUs from Pampa del Indio [11, 12].

In the present study, parasite load was directly associated with host infectiousness in all domestic and sylvatic host species, and may be considered a surrogate of infectiousness. At similar levels of parasite load < 10 Pe/ml, domestic hosts showed a significantly lower infectiousness than the main sylvatic hosts (Fig. 3). Heterogeneous parasite distribution among tissues, as in *Leishmania* infected-dogs and rodents [28, 54], or intrinsic differences in growth rates among parasite DTUs may explain these differences in host infectiousness. Even though triatomine molting rates were not significantly associated with infectiousness in the GLM analysis, it was twofold higher for *D. albiventris* and *D. novemcinctus* than for domestic host species (20% vs 10%) within the range of 1–10 Pe/ml. Therefore, the observed differences in infectiousness between domestic and sylvatic hosts within this range of parasite loads may in part be attributed to the larger blood intake of sylvatic mammals.

We fitted a non-linear model to the relationship between infectiousness and bloodstream parasite load, as this relationship was previously determined for other vector-borne diseases [4, 55, 68]. Model 1 exhibited the best fit for both host groups, achieving the lowest AIC value. Interestingly, Model 1 includes components related to vector competence through parameter B1 that are absent in the sole quantification of parasite load by qPCR as a proxy of reservoir host competence. The B2 parameter was twofold higher in sylvatic hosts than in domestic hosts, which agrees with differences in the infectiousness curves of domestic and sylvatic hosts (Fig. 3). We estimated the B2 value that would result from empirical data combining the mean blood meal volume of *T. infestans* fourth-instar nymphs (0.18 ml, Cerisola et al. [69]) and the minimum number of parasites required to infect a vector (one parasite) (Moll-Merks et al. [70]), resulting in a parameter value of 0.18. This value is similar to the one estimated by our non-linear model for the domestic hosts’ dataset (0.17). The parameter B1, associated with the probability that a vector becomes infected, had similar values in sylvatic and domestic host models and is close to the maximum value. Both the establishment and development of *T. cruzi* within triatomines may be affected by the gut microbiota [71–73] and by digestive enzymes, agglutinins, hemolysins and antimicrobials [74]. The modeling approach employed in the present study would allow the study of reservoir host competence in a larger number of host species, thereby avoiding examination through xenodiagnosis which is more time-consuming. Several mathematical models have been developed to model *T. cruzi* transmission dynamics; however, most of these have omitted variations in infectiousness among hosts [75–78] or only incorporated a mean infectiousness per host species [79]. Therefore, incorporating infectiousness variability from the distribution of parasitemia within the host population and vector-host contact probabilities (through blood-feeding patterns) allowed us to assess the relative contribution of different host groups to the transmission of *T. cruzi*.

Our results are based on the same protocols for xenodiagnosis, DTU identification and quantification of parasite loads, which allows a valid comparison of outcomes among host species. Although we used two protocols for xenodiagnosis (artificial in humans and natural for other hosts), the results of artificial xenodiagnosis were similar to those obtained when the vector directly fed on humans in other studies [12]. Our inferences are restricted to the Gran Chaco region as few individuals were from Misiones. Due to the small number of samples, to model infectiousness we grouped hosts by transmission cycle instead of studying each species separately. Even though the observed curve of infectiousness in relation to parasite load (Fig. 3) supports the grouping criteria, the inclusion of more samples for some host species may allow modeling the responses separately for each species. Human samples were biased towards early chronic patients since individuals aged < 21 years were eligible for etiological treatment and prioritized for serodiagnosis [12]. Infants aged < 9 months were excluded from the serosurvey; when infected with *T. cruzi* infants in this age group have high parasite loads [80].

## Conclusions

In conclusion, the wide variability and over-dispersed distribution of *T. cruzi* bloodstream load across sylvatic and domestic host species determined in our study appears to be shared with other multi-host parasites that show a broad genetic diversity [81–83]. Our results have implications for further understanding the epidemiology of Chagas disease and the application of control measures since the heterogeneous distribution of parasite load may affect transmission dynamics by increasing *R*_0_ and the efforts required to reduce the infection in humans. Addressing the issue of whether there is a higher contact rate between triatomines and the small fraction of domestic hosts exhibiting the highest parasite loads would provide support to the development of novel and cost-effective Chagas disease transmission control strategies.

## Supplementary Information

**Additional file 1: Table S1.** Profile of examined hosts according to species.

**Additional file 2: Dataset S1.** Results per individual (Database).

## Data Availability

The datasets supporting the conclusions of this article are included in the article (and its additional files).

## Abbreviations

DTU: Discrete typing units
GEB: Guanidine–EDTA blood samples
kDNA-PCR, SAT DNA-PCR: PCR amplification of the minicircle of kinetoplast DNA and satellite DNA, respectively
IAC: Internal amplification control DNA
Pe: Parasite equivalents
qPCR: Quantitative PCR
