# Supplementary material for: Over-dispersed Trypanosoma cruzi parasite load in sylvatic and domestic mammals and humans from northeastern Argentina
Source: Parasit Vectors. 2022 Jan 24;15:37. doi: 10.1186/s13071-022-05152-7 (PMC8785451; doi:10.1186/s13071-022-05152-7)
Supplement: Supplementary file 1 — Additional file 1: Table S1. Profile of examined hosts according to species. [file 13071_2022_5152_MOESM1_ESM.docx]

**Supplementary Table 1.** Profile of examined hosts according to species.

| Host species | Province | Prevalence of infection % (No. examined) | Mean infectiousness % | Only for hosts examined by qPCR | | | | | | | | |
| --- | --- | --- | --- | --- | --- | --- | --- | --- | --- | --- | --- | --- |
|  |  |  |  | Number examined by qPCR (number infected hosts) | Median age | Sex ratio (male: female) | No of individuals with DTU identified (%) | | | | | Reference of host samples |
|  |  |  |  |  |  |  | TcI | TcIII | TcV | TcVI | Median parasite load (IQR) |  |
| *Didelphis albiventris* | Misiones | 16 (55) | 62 | 4 (9) | Na | 1:00 | 4 (100) | 0 (0) | 0 (0) | 0 (0) | 52.5 (2.8-72.0) | [36] |
| *Didelphis albiventris* | Chaco | 38 (42) | 56 | 9 (16) | Na | 2:01 | 6 (100) | 0 (0) | 0 (0) | 0 (0) | 10.8 (0.1-38.0) | [13] |
| *Dasypus novemcinctus* | Chaco | 58 (26) | 74 | 10 (16) | Na | 1:01 | 0 (0) | 9 (100) | 0 (0) | 0 (0) | 38.3 (6.2-149.4) | [13] |
| [*Chaetophractus vellerosus*](https://es.wikipedia.org/wiki/Chaetophractus_vellerosus) | Chaco | 6 (16) | 35 | 1 (1) | Na | 1:00 | 0 (0) | 1 (100) | 0 (0) | 0 (0) | 40 (NA) | [13] |
| [*Tolypeutes matacus*](https://es.wikipedia.org/wiki/Tolypeutes_matacus) | Chaco | 13 (16) | 8 | 1 (2) | Na | 1:00 | 0 (0) | 1 (100) | 0 (0) | 0 (0) | Nd | [13] |
| [*Euphractus sexcinctus*](https://es.wikipedia.org/wiki/Euphractus_sexcinctus) | Chaco | 20 (5) | 0 | 2 (2) | Na | 1:04 | 0 (0) | 1 (50) | 0 (0) | 0 (0) | 2.1 | [13, 38] |
| [*Conepatus chinga*](https://es.wikipedia.org/wiki/Conepatus_chinga) | Chaco | 100 (1) | 100 | 1 (1) | Na | 1:00 | 0 (0) | 1 (100) | 0 (0) | 0 (0) | 7 (NA) | [38] |
| *Felis catus* | Chaco | 29 (87) | 49 | 13 (15) | 1 | 2:01 | 2 (17) | 0 (0) | 2 (17) | 8 (66) | 9.7 (2.9-96.9) | [11, 84] |
| *Canis lupus familiaris* | Chaco | 23 (698) | 48 | 70 (78) | 4 | 2:01 | 1 (3) | 3 (7) | 5 (12) | 32 (78) | 5.5 (5.4-21.9) | [11, 22, 84] |
| Humans | Chaco | 25 (3578) | 5 | 88 (895)^a^ | 15 | 1:01 | 0 (0) | 0 (0) | 20 (91) | 2 (9) | 0.2 (0.0-0.5) | [12] |

Na. Does not apply.

Nd. DNA not detectable by qPCR.

a. Eleven seropositive humans were included but not examined previously by qPCR in Macchiaverna et al. (12).
